# Observation and research of deep underground multi-physical fields—Huainan −848 m deep experiment

**DOI:** 10.1007/s11430-022-9998-2

**Published:** 2022-12-19

**Authors:** Yun Wang, Yaxin Yang, Heping Sun, Chengliang Xie, Qisheng Zhang, Xiaoming Cui, Chang Chen, Yongsheng He, Qiangqiang Miao, Chaomin Mu, Lianghui Guo, Jiwen Teng

**Affiliations:** 1 School of Geophysics and Information Technology, China University of Geosciences, Beijing, 100083 China; 2 School of Geophysics and Measurement-control Technology, East China University of Technology, Nanchang, 330013 China; 3 Innovation Academy for Precision Measurement Science and Technology, Chinese Academy of Sciences, Wuhan, 430077 China; 4 Institution of Engineering Protection, IDE, AMS, PLA, Luoyang, 471023 China; 5 State Key Laboratory of Mining Response and Disaster Prevention and Control in Deep Coal Mines, Anhui University of Science and Technology, Huainan, 232001 China; 6 Institute of Geochemistry, Chinese Academy of Sciences, Guiyang, 550081 China

**Keywords:** Multi-physical fields, Radioactivity, Gravity, Geomagnetic, Electromagnetic, Earthquake, Observations deep underground

## Abstract

Compared with the surface, the deep environment has the advantages of allowing “super-quiet and ultra-clean”-geophysical field observation with low vibration noise and little electromagnetic interference, which are conducive to therealization of long-term and high-precision observation of multi-physical fields, thus enabling the solution of a series of geoscience problems. In the Panyidong Coal Mine, where there are extensive underground tunnels at the depth of 848 m belowsea level, we carried out the first deep-underground geophysical observations, including radioactivity, gravity, magnetic, magne-totelluric, background vibration and six-component seismic observations. We concluded from these measurements that (1) the background of deep subsurface gravity noise in the long-period frequency band less than 2 Hz is nearly two orders ofmagnitude weaker than that in the surface observation environment; (2) the underground electric field is obviously weaker thanthe surface electric field, and the relatively high frequency of the underground field, greater than 1 Hz, is more than two orders of magnitude weaker than that of the surface electric field; the east-west magnetic field underground is approximately the same asthat at the surface; the relatively high-frequency north-south magnetic field underground, below 10 Hz, is at least one order ofmagnitude lower than that at the surface, showing that the underground has a clean electromagnetic environment; (3) in additionto the high-frequency and single-frequency noises introduced by underground human activities, the deep underground spacehas a sig-nificantly lower background vibration noise than the surface, which is very beneficial to the detection of weakearthquake and gravity signals; and (4) the underground roadway support system built with ferromagnetic material interferesthe geomagnetic field. We also found that for deep observation in the “ultra-quiet and ultra-clean” environment, the existinggeophysical equipment and observation technology have problems of poor adaptability and insufficient precision as well asdata cleaning problems, such as the effective separation of the signal and noise of deep observation data. It is also urgent tointerpret and comprehensively utilize these high-precision multi-physics observation data.

**Electronic Supplementary Material:**

Supplementary material is available in the online version of this article at 10.1007/s11430-022-9998-2.

## 1. Introduction

The continuous observation of multi-physical fields from Earth and extraterrestrial bodies is not only important for understanding the formation and evolution of Earth and its relationship with the universe, but it is also important for human survival, disaster prevention and reduction. It also meets the objective needs of human economic and social development to continuously explore resources and environmental opportunities (Argo et al., 1995; Lu et al., 1999). Moreover, the long-term, stable and networked observation of basic geophysical fields such as gravity, geomagnetic, electromagnetic and seismic provides essential information. Since the founding of the People's Republic of China, China has successively established and improved Earth-based and space-based station networks for investigating gravity, geomagnetism, electromagnetism and earthquakes, forming a geophysical observation network throughout Chinese mainland, which has gradually expanded to ocean observations in recent years (Wang, 2007; Hao et al., 2019; Wei et al., 2019) to enable the global coverage of multi-parameter observations of geophysical fields.

However, with the continuous and rapid development of China's economy and society, the conditions of sound, light, electricity, and magnetism required for multi-physical field observations of the Earth have deteriorated sharply (Bleier and Freund, 2005), resulting in a decrease in the reliability of observation data. This challenge has seriously affected related research and scientific and technological support capabilities. With the large-scale development of land resources in recent years, the surface capacity has approached its limit (He, 2016), and the development of underground space has become essential in many fields. Although different industries and fields have their own definitions of deep underground space (Teng et al., 2016, 2017), proceeding deep into the earth is a strategically scientific and technological problem that we must solve (Xi, 2016). In particular, deep underground multi-physical field observation has become one of the most important frontier topics in Earth science research.

Compared with China's establishment of multi-physical field observation networks only in land areas and certain other key areas (Ye et al., 2010), developed countries such as Japan and the United States, as well as the European Union, have established relatively complete joint observation networks in ocean and land areas and carried out underground and deep underground multi-physical field observations. For example, as superconducting gravimeters have extremely low instrument noise and require an ultraquiet environment (Ma D et al., 2019), the Membach Laboratory in Belgium (40 m underground), the Walferdange underground laboratory in Luxembourg (80 m underground), the Kamioka underground laboratory in Kamioka, Japan (the inner cavity of a mountain, covered approximately 1000 m), and the LSBB underground laboratory in France (covered approximately 550 m) have carried out surface and deep underground experiments to perform high-precision superconducting gravity and geomagnetic observations (Waysand et al., 2009). The observation quality of the superconducting gravimeter in the French LSBB laboratory exceeds that of the most accurate stations in the world, showing that the deep subsurface environment has a very low level of noise (Rosat et al., 2018). Geomagnetic data from more than 220 surface stations around the world are also subject to severe man-made electromagnetic noises, the quality of the electromagnetic observations is poor, and time-varying electric fields are generally not recorded (Fraser-Smith and Helliwell, 1985; Galejs, 1972). Only the French LSBB laboratory has carried out experiments related to continuous observations of deep underground electromagnetic fields and earthquakes (Wang et al., 2011). The United States carried out comprehensive geophysical observations of 4000-meter-deep wells on the San Andreas fault (Chavarria et al., 2003), and Japan established a geophysical observation network based on dozens of kilometers of boreholes for earthquake prediction research (Fujimoto et al., 2007). The Federal Republic of Germany used continental deep drilling to carry out water injection-induced seismic experiments in 4000-meter-deep boreholes (Tu and Chen, 2002). The DUSEL Deep Earth Laboratory in the United States measured vibration and noise at different depths at the beginning of the project construction and found that at a depth of 2000 feet, the horizontal vibration noise is one order of magnitude lower than that at the surface, and the high-frequency band (≥1 Hz) of vertical vibration is also reduced by an order of magnitude (Lesko, 2009).

Compared with the surface, the deep underground has obvious advantages in observation environments (Marfaing et al., 2009; Nishijima et al., 2013), and the utilities of the "ultraquiet" and "ultraclean" environment of deep underground space can support high-precision four-dimensional observation of multi-physical fields. China has been actively exploring deep observation since the end of the last century. Since the scientific drilling of the East China Sea in Jiangsu in 2001 (Gao and Jin, 2001), national deep observations have mainly been based on scientific drilling wells, in which the long-term physical field observations carried out include fluid pressure, strain, well temperature and short-period earthquakes (Wang et al., 2006; Xu et al., 2016; Zeng et al., 2020). To prevent the earth resistivity observations from being contaminated by surface interferences, the China Earthquake Administration successively built more than 10 shallow well-ground resistivity stations since 2010, including the Haian station in Jiangsu and the Daboshe station in Hebei (Nie et al., 2010; Yang et al., 2012; Mao et al., 2014; Wang et al., 2015). Seismic observations of a 1300-meter borehole on the Chelongpu fault were carried out in Taiwan Province, China (Ma et al., 2012). However, due to factors such as the narrow borehole space and the lack of supporting high-precision instruments, the high-precision geomagnetic, gravity, seismic, electromagnetic and other multi-parameter four-dimensional observations in deep wells are basically still in the early stage; even for deep cavities with better accessibility, due to the high maintenance cost of the deep environment, only small-scale observation experiments and seismic observations in caves, tunnels, and air-raid shelters have been conducted (Li et al., 2018).

Using the 848 m deep underground tunnels in the Huainan Coal Mine (Huainan Deep Underground Lab, HDUL), we carried out the first deep multi-physics joint observation in early 2020 and obtained a meaningful and effective observation dataset. In this paper, a general introduction to this observation and the preliminary analysis of the data are provided, the characteristics of deep observation and its similarities and differences with surface observation are summarized, the development of deep scientific observation is promoted, and the key scientific and technological problems in related fields based on deep underground observations are explored and discussed.

## 2. The first observation in the HDUL

The Panyidong Coal Mine, with an elevation of 22 m, belonging to Huaihe Energy Company Limited, is located in Huainan city, Anhui Province, and lies on the north bank of the Huaihe River, more than 100 kilometers away from the TanLu fault zone in the east and approximately 200 km away from the junction of the South China Block and the North China Block in the south (Deng et al., 2003; Wessel et al., 2019), as shown in Figure 1(a). Panyidong is a large-scale coal mine designed and constructed according to the modern mechanized standard in 2012. For the purpose of coal mining reduction, the Panyidong Coal Mine was shut down in 2018. There are still complete production and living facilities on the surface to ensure the power supply and safety of the mining area underground. To distinguish cavities usually located in mountains that are characterized by covering thickness, the absolute height above sea level is used in this paper to represent the depth of the underground laboratory, and the negative value represents the depth of the laboratory below sea level. Many crisscrossed tunnels are built at depths of −848 m and −1042 m and supported by concrete, as shown in Figure 1(b), with sections (width×height) of 5 m×6 m or 3 m×4 m. These tunnels have a total length of approximately 40 kilometers, which provides more than 800,000 cubic meters of space underground. Due to the intermittent operation of fans at the wellhead of the surface, the temperature and humidity of the underground tunnels, especially near the underground wellhead, change seasonally similar to those at the surface, with the annual temperature and humidity ranging from 13°C to 35°C and 45% to 85%, respectively. The main tunnels, as shown in Figure 1c and 1d, with wind speeds of 3.0 to 4.5 m $s^{-1}$, are especially suitable for human access and long-term instrument deployment and have the basic conditions necessary for multi-physical field observations.

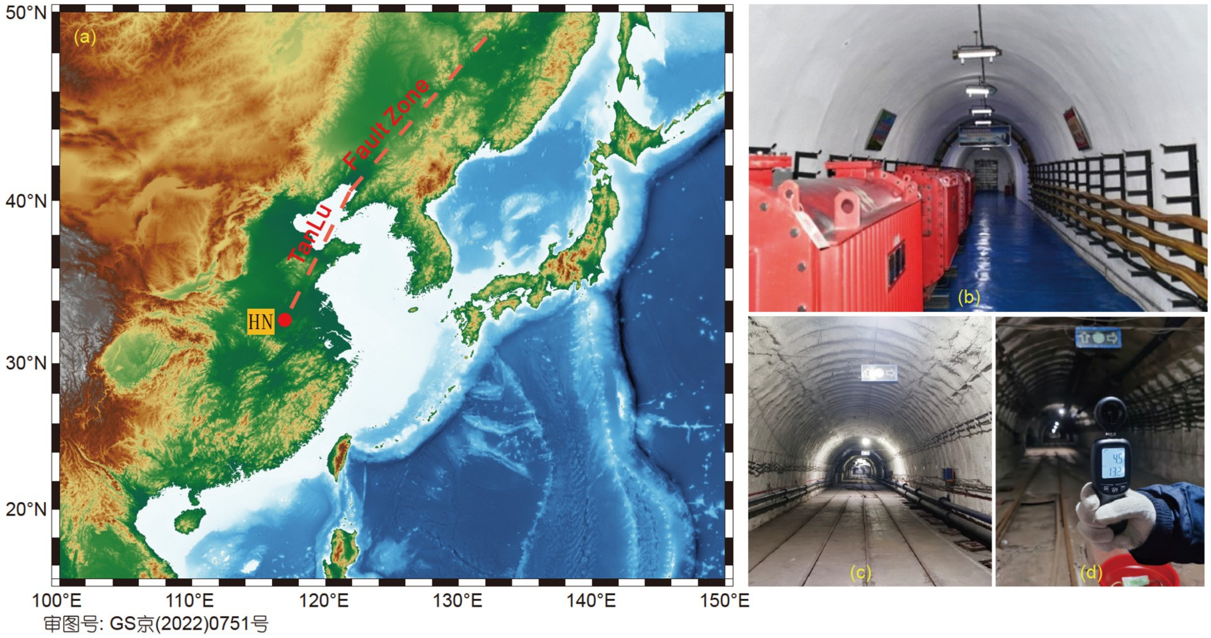

**Figure 1** HDUL: (a) the location of the lab, (b) a branch tunnel underground, (c) the main tunnel 848 m underground, and (d) measurements of the wind speed and temperature in the main tunnel.

### 2.1 Surface and deep underground observations

The geophysical observations were executed in horizontal tunnels 848 m underground and included gravity, geomagnetic, electromagnetic, seismic, microseismic, and radioactivity surveys, accompanied by surface observations, to identify the background noise level and radioactivity deep underground. Since January 17, 2020, more than 30 sets of geophysical instruments have been deployed, as shown in Figure 2, with more than 3 months of observation. Three-component rotational seismometers and three-component translational seismometers were set up on the surface and in the deep underground tunnel, respectively, and at the same time, a differential translational seismometer array was set downhole for comparison and analysis. It should be noted that the three-component rotational seismometer R2 not only records the rotation rates of three axes but also the inclination and acceleration in three directions. Two sets of three-component microseismometers in a triangular array were placed on the surface and underground. One magnetometer was placed on the surface, and the other two were placed underground, where a steel-sealed escape trunk and reinforced concrete were chosen as the observation sites for surveying the underground magnetic interference. Gravity was also measured on the surface and in the underground tunnel but with different types of gravimeters. Many radioactivity measurements were performed, including water and gas radon and gamma and neutron fields. The multi-physical observation system, instrument parameters, observation date and duration are shown in Tables 1 and 2, and some earthquake events during the observation are shown in the Appendix (https://link.springer.com).

To ensure the accessibility of the deep underground space, fans and the underground power supply were running during the observations. In addition, daily inspections were carried out to ensure the safety of the deep underground observations, requiring the elevator (cage) to operate on a regular basis. These operating electromechanical devices undoubtedly impacted the multi-physical observation. Surveying the artificially generated noise was also an important task of these geophysical observations for the design and construction of the HDUL. Therefore, during the observations, we investigated vibration and electromagnetic interference.

### 2.2 Observation results and analyses

*2.2.1 Radioactivity survey*

To determine whether the HDUL has a radioactive environment that is safe for people to stay in for a long time, we investigated the background level of cosmic rays to provide background parameters for deep underground experiments related to high-energy physics and chemistry.

(1) Radon measurement. The radon measurement was carried out on the surface and in the −848 m tunnel. Surface measurements showed that the radon concentration in the air was between 5 and 35 Bq $m^{-3}$, and the outdoor environment had a significantly lower concentration than the indoor environment. In tunnels deep underground, the main tunnel with good ventilation, the branch tunnel with low wind speed, and the rock outcrops were chosen for observation and comparison, as shown in Figure 3. The radon concentration in the underground tunnel was higher than that on the surface, approximately 10 to 100 Bq $m^{-3}$, and the average value in the tunnel with good ventilation was approximately 40 Bq $m^{-3}$, while the average value in the branch tunnel reached up to 69–70 Bq $m^{-3}$. However, the air radon level of the −848 m tunnel was lower than the national limit of 150 Bq $m^{-3}$ defined for the class of civil buildings (GB50325-2020), which is a safe residential environment.

Groundwater samples were collected at two locations underground, and water radon was measured in the laboratory. The mean radon concentration in the seepage tank was 283 Bq $m^{-3}$, and the mean radon concentration of the fresh seepage was 2552 Bq $m^{-3}$. While water radon deep underground presented a relatively considerable concentration, it was still at a low level compared with observations of shallow water before earthquakes occur (Zhao et al., 2016; Huang et al., 2019; Su et al., 2020).

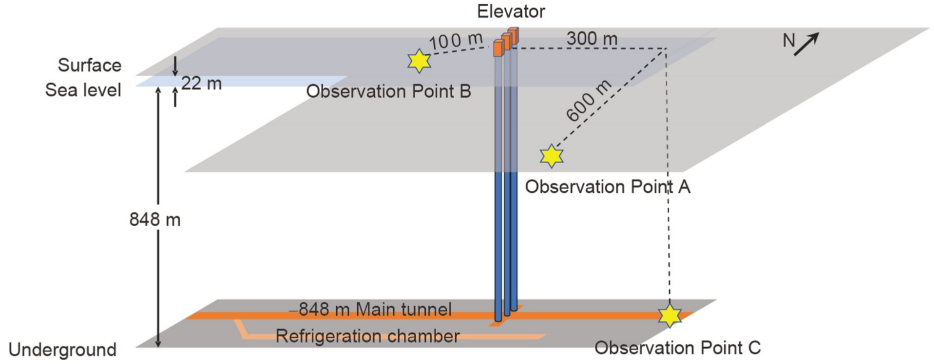

**Figure 2** Layout of the observation system in the HDUL.

**Table 1** Instruments and their parameters deployed in the first multi-physical field observations

| Equipment | Main performance | Observation system |
|---|---|---|
| Rotational seismometer<br>R-2 (2 sets) | Frequency range: 0.033–50 Hz<br>Manual nominal resolution: $6\times10^{-8}$ rad $s^{-1}$ (@1 Hz)<br>Dynamic range: 117 dB<br>Self-noise: –125 dB (rel. 1 rad $s^{-2}$ $Hz^{-1/2}$) | 1 set each on the surface and underground |
| Broadband Seismometer: CMG-40TD (4 sets) | Frequency range: 30 s–100 Hz<br>Self-noise: 20 s–16 Hz lower than NLNM | 3 sets in the −848 m tunnel, 1 set on the surface |
| Magnetometer<br>GSM-19T Proton Magnetometer (3 sets) | Sensitivity: 0.15 nT (1 s sampling interval), 0.05 nT (4 s sampling interval)<br>Resolution: 0.01 nT | 1 set of surface:<br>Point number 4448, sampling interval 300 s<br>2 sets underground:<br>Point number 4451 (tunnel), sampling interval 5 s; Point number 4473 (closed chamber), sampling interval 300 s |
| Microseismometer:<br>OMNI-2400 three-component geophone (4 sets)<br>PS-1 low-frequency three-component geophone (2 sets) | Band range:<br>15–500 Hz (OMNI-2400)<br>4–800 Hz (PS-1)<br>Sensitivity:<br>0.52±5% @80 Hz (OMNI-2400)<br>0.062@5 Hz (PS-1) | 3 sets of surface and underground |
| Electromagnetic instruments:<br>MTU-5A (2 sets)<br>LEMI-417 digital seven-component magnetotelluric station (1 set) | Frequency range:<br>DC−10 kHz (MTU-5A),<br>DC−0.3 Hz (LEMI-417) | 1 set of broadband for surface and tunnel;<br>Long-term arrangement on the surface |
| High precision gravimeter:<br>Burris and LCR-ET20 | Observation accuracy: 0.1 Gal | 1 set in a quiet room on the surface<br>1 set in the −848 m tunnel |
| Radioactivity observation instrument:<br>$\gamma$ spectrometers (6 sets), including BH3013B X-$\gamma$ radiation dose rate meter and high-purity germanium spectrometer, etc.<br>Radon detectors (5 sets), including RAD-7, AlphaGUARD PQ200, etc.<br>Neutron measuring device (3 units), including neutron counter NeutronRAEII, etc. | Performance parameters of BH-3013B Portable X-$\gamma$ Radiation Dose Rate Meter<br>Energy response: 25 keV−3 MeV, the limit of variation is ±15%<br>Range: $(1–10000)\times10^{-8}$ Gy $h^{-1}$<br>Inherent error: no more than 10%<br>performance parameters of RAD-7 Radon detector:<br>Detection range: 4–750,000 Bq $m^{-3}$ | Combination of point and line observation, including rock and water sampling and laboratory analysis |

**Table 2** Period and duration of the first multi-physical observation

| Observation point | Gravity | Geomagnetism | Broadband magnetotelluric | Long period magnetotelluric | Translational displacement /velocity | Rotational rate | Tilt | Microseism |
|---|---|---|---|---|---|---|---|---|
| Ground | 19 Jan to 15 May, 118 days | 17 Jan to 15 March, 58 days | 17 Jan, 20 hours | 17 Jan to 16 April, 90 days | 18 Jan to 1 Feb, 14 days | 20 Feb to 22 Feb, 2 days | 20 Jan to 22 Jan, 2 days | 17 Jan, 12 hours |
| Underground | 18 Jan to 26 Jan, 9 days | 17 Jan to 26 April, 100 days | 17 Jan, 20 hours | | 19 Jan to 2 Feb, 14 days | 30 March, 2 hours | 30 March to 15 May, 45 days | 17 Jan, 12 hours |

(2) Gamma spectroscopy and neutron dose rate measurement. At locations with poor and good ventilation, a portable high-purity germanium high-resolution gamma spectrometer was used to record $\gamma$ counts for 58,829 and 21,348 s, respectively. The results, indicated by the black curve shown in Figure 4, for the measurements in the No. 5 tunnel with poor ventilation reveal that the $\gamma$ count per second in the deep underground tunnel is very low. Compared with the red curve, data from the Jinping underground laboratory (Zeng et al., 2014; Cheng et al., 2017), the background level of the $\gamma$-radiation in the HDUL is comparable or slightly better.

We prospected the neutron dose rate in two survey lines and found that the total neutron equivalent dose in 3 h is 0.04 μSv, a very small value, which indicates that the overlying layer of nearly 870 m provides an effective shield from cosmic rays.

(3) Rock radioactivity. The content of radioactive elements in coals and surrounding rocks have an important influence on the radioactive background of coal mine tunnels. Many studies have analyzed the content of radioactive elements in coals and their surroundings, such as uranium, thorium, and potassium, as well as surrounding rocks of the Jinping Lab (Huang et al., 2001; Wang et al., 2004; Liu et al., 2018). As shown in Table 3, in which the radioactive element contents of the Jinping and Huainan labs are compared, they are at

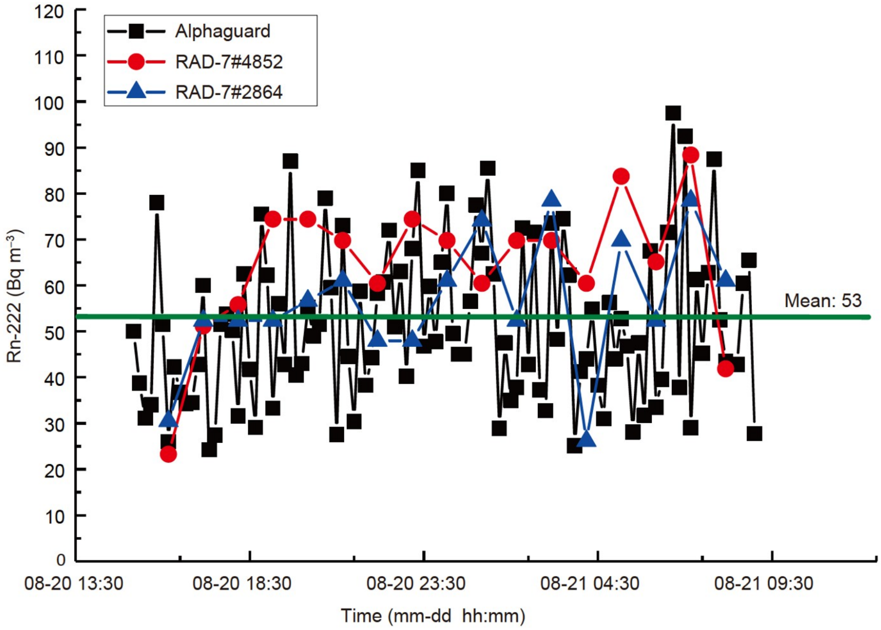

**Figure 3** Radon concentration at Chamber No. 5 in a maintenance tunnel 200 m in length (which presents poor ventilation). The black, red and blue dotted lines in the figure present recordings of the Alpha PM and RAD7, respectively, of which 4852 and 2864 are the numbers of the two RAD7 devices.

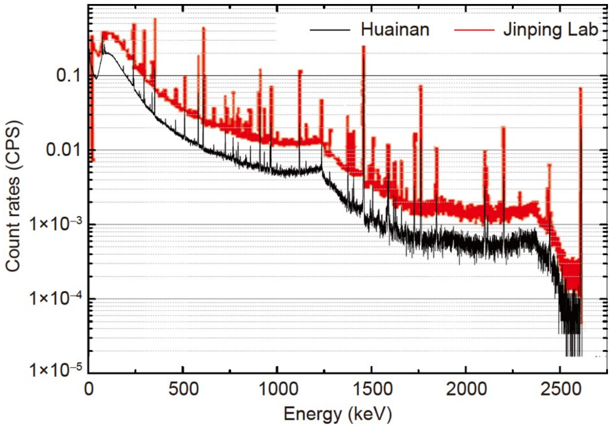

**Figure 4** Gamma spectrum measurement in the HDUL (black line) and at the Jinping laboratory (red line) (red data from Zeng et al., 2014).

similar levels, with those in the Jinping lab at a relatively lower level because the surrounding rocks in the Jinping lab are marine carbonate and marbles.

### 2.2.2 Gravity observation

Two spring gravimeters were used in this observation to evaluate the gravity noise level. As introduced in the paragraphs above, there are many possible noise sources, such as fans, water pumps, elevators (cages), transformers, high-voltage power lines and railways, in underground tunnels, which may contaminate gravity signals on the high-frequency band greater than approximately 1 mHz.

Figure 5 presents a comparison of the original gravity recordings between the surface and the −848 m tunnel. It can be concluded that the continuous gravity observations are reliable, the time-variant trends are consistent, and the comparison of the gravity noise underground and at the surface should be credible. As illustrated in Figure 6, the noise deep underground at the frequency band of 2–10 Hz is stronger than that at the surface, while the low-frequency band reverses and is more than 2 orders of magnitude lower than that at the surface. It can thus be inferred that the HDUL would be suitable for low-frequency high-precision gravity observation (Zhang et al., 2021).

### 2.2.3 Geomagnetic observation

Three proton-precession magnetometers were used for the geomagnetic observation, one on the surface, named No. GSM-19T-4448, and two on the subsurface, named No. GSM-19T-4451 and 4473. To survey typical magnetic disturbances in the HDUL, the magnetometer numbered GSM-19T-4451 was located at the end of one tunnel, surrounded by concrete walls, and disturbed by various types of magnetic noises, such as power supply, fans, drainage facilities and metal support structures. The magnetometer numbered GSM-19T-4473 was located in the steel-sealed cabinet, which provided some degree of geomagnetic shielding. Figure 7 shows the geomagnetic recordings of the three sites.

According to the WMM (2019–2024) model (magnetic field calculator, provided by NOAA: https://www.ngdc.noaa.gov/geomag/calculators/magcalc.shtml#igrfwmm), the average total magnetic field on the surface of Huainan is approximately 50691 nT, which increases to approximately 50746 nT at a depth of 2000 m underground. The real average magnetic field on the surface is approximately 45829 nT, and the average magnetic field at site 4451 un-

**Table 3** Comparison of U/Th/K content between Huainan and Jinping[a)]

| Location | U-238 (Bq kg$^{-1}$) | Th-232 (Bq kg$^{-1}$) | K-40 (Bq kg$^{-1}$) |
|---|---|---|---|
| Jinping | 3.69–4.21 | 0.52–0.64 | 4.28 |
| Huainan | 2.21–14.892 | 6.84–14.652 | 6.252 |

a) Jinping data from Wang et al. (2004)

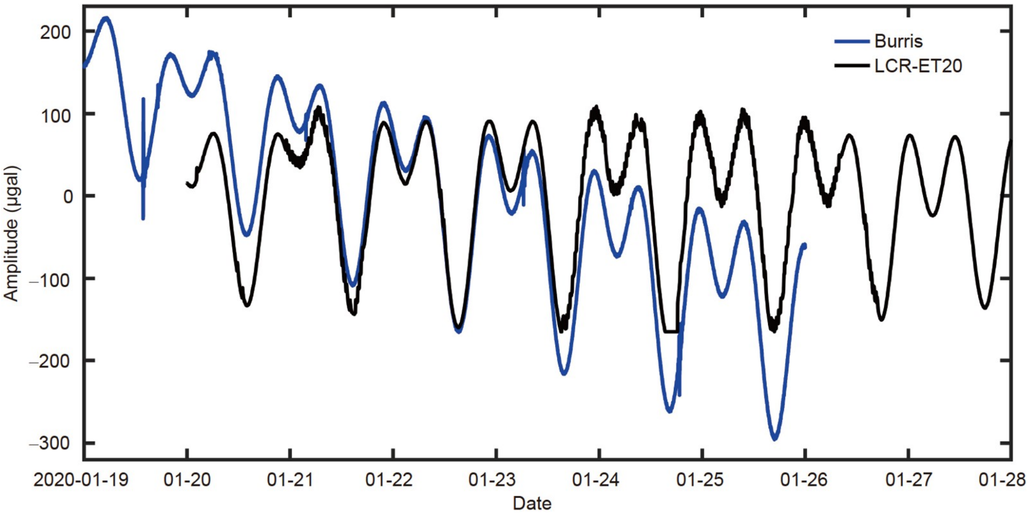

**Figure 5** Comparison of the original gravity data on the surface (the black curve) and in the −848 m tunnel (the blue curve). There is a crossover of two curves caused by different drifts of the two different gravimeters, as the original data in this picture have not been corrected (Zhang et al., 2021).

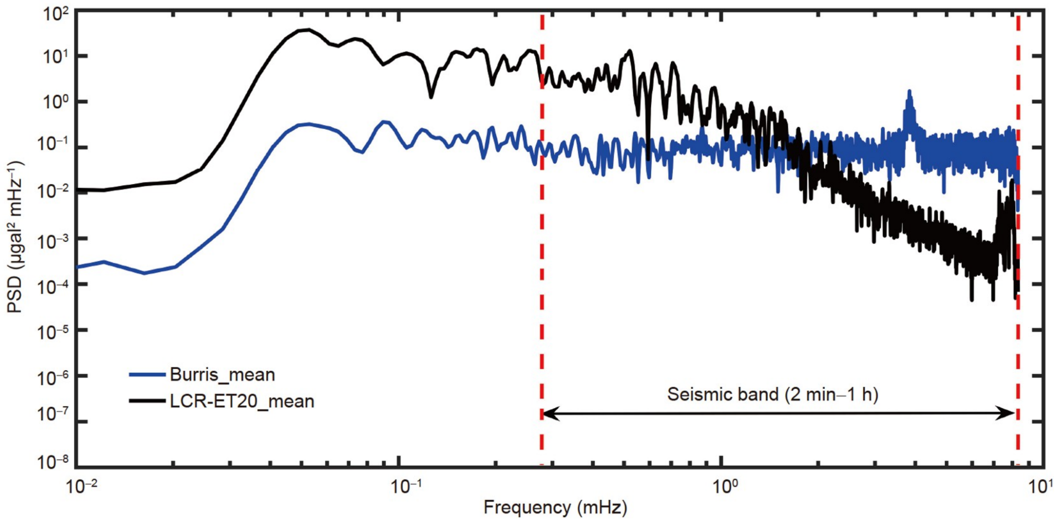

**Figure 6** Average power spectrum of Burris (underground) and LCR-ET20 (surface) gravimeters (Zhang et al., 2021).

derground is 93525 nT, indicating that the reinforced concrete support structure or the surrounding interference has seriously affected the observation, magnifying the results nearly 2-fold. The average magnetic field at site 4473 is approximately 28413 nT, indicating that the thick steel plate of the support structures provides a shield and attenuates half of the geomagnetic intensity. Therefore, we can conclude that the interference of ferromagnetic material from the support structures and their induced magnetic field should be given special attention in underground geomagnetic observation.

### 2.2.4 *Electromagnetic observation*

According to the requirements of magnetotelluric (MT) observation, we set the surface and underground stations in "L" mode. The electrode's distance was approximately 50 m in

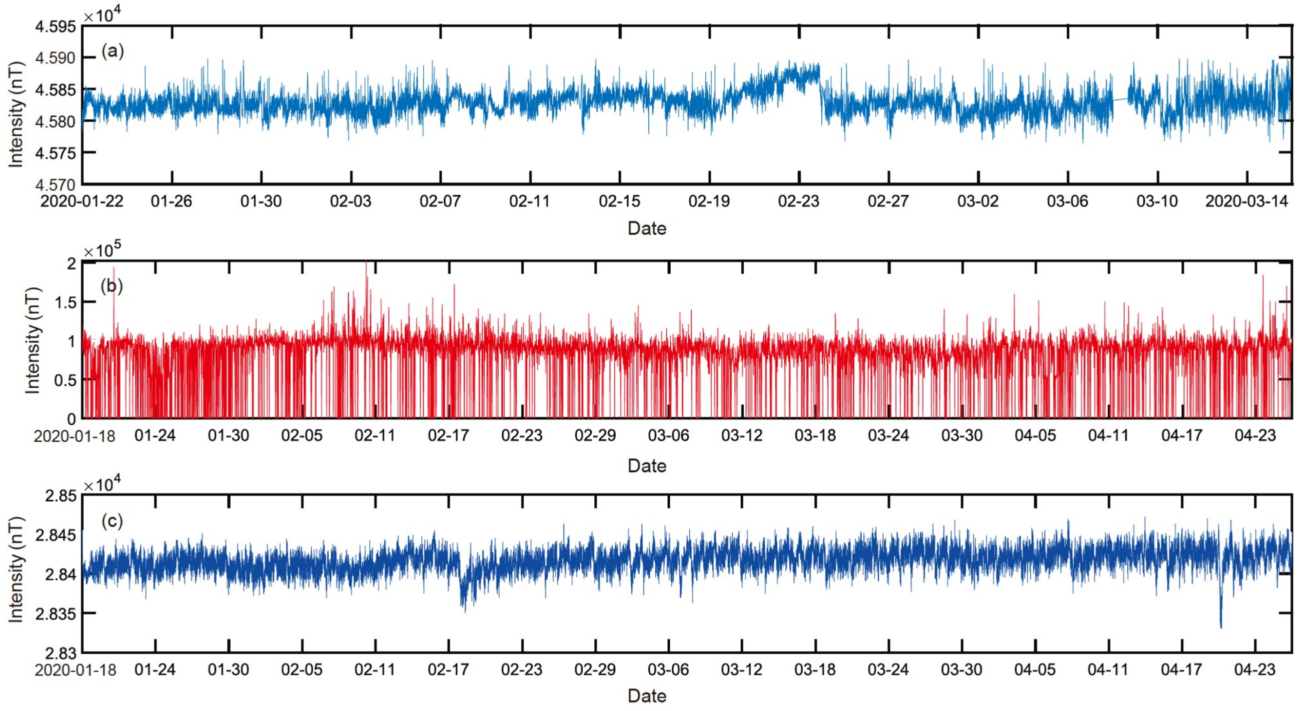

**Figure 7** The original geomagnetic observation, of which (a) is surface No. 4448, (b) is underground No. 4451 and (c) is No. 4473.

both the north-south (NS) and east-west (EW) directions. On the surface, iron frame brackets, transformers, and power lines generate strong noise in electromagnetic (EM) signals. In the underground location, in addition to the high- and low-voltage power lines, reinforced concrete supports and railways will also cause considerable noise. We employed clay bedding, burying, and watering to meet the requirement of grounding resistance for electrodes, since the tunnels are covered with reinforced concrete and there is no way to bury electrodes and magnetic coils.

Sampling rates of 2400, 150, and 15 Hz were set in the MT observations. The data were recorded continuously with a 15 Hz sampling rate and recorded periodically with the two other sampling rates. Figure 8 shows the comparison of the 12-hour EM time series recorded synchronously on the surface and underground, in which the sampling rate was set as 15 Hz and the data were mean removed. The MT observation indicates that (1) the surface electric field is affected by apparent periodic noise, while the underground data are 'cleaner'. Limited to the concrete tunnel, the non-polarizable electrodes could not be buried well, and the grounding resistance increased rapidly with water loss. The unstable grounding resistance probably induced the obvious drift of the underground electric field. (2) The NS horizontal magnetic fields ($B_x$) on the surface and underground are relatively close, while the EW magnetic fields ($B_y$) are significantly lower than the normal magnetic field, which needs further verification.

To further quantify the background noise level of the surface and underground environments, we calculated the power spectral density (PSD, Figure 9) and found the following characteristics.

(1) Electric field: The electric field underground is lower than that at the surface. In the high-frequency band above 10 Hz, the PSD of the surface electric field is generally greater than $10^{-1}$(mV km$^{-1}$)$^2$ Hz$^{-1}$, while it is generally close to $10^{-5}$(mV km$^{-1}$)$^2$ Hz$^{-1}$ underground. There are nearly two orders of magnitude differences between surface and underground observations. For the low-frequency band below 10 Hz, the difference between the surface and underground decreases gradually and approaches the same level after 0.1 Hz.

(2) Magnetic field: The NS magnetic field is much higher than that of EW data, and the difference is nearly two orders of magnitude. In the high-frequency band above 6 Hz, the surface NS magnetic field ($B_x$) is higher than that underground, and the difference is nearly one order of magnitude above 10 Hz. The EW magnetic field ($B_y$) of the surface and underground is basically at the same level.

(3) In the middle- and high-frequency bands (above 50 Hz), the observations are contaminated by multiple EM noises, especially at frequencies of 50 and 100 Hz.

#### 2.2.5 *Microseismic ambient noise observation*

Because gravity observations mainly consider ambient noise in the relatively lower frequency band, we supplemented vibration and noise surveys in the middle- and high-frequency bands. The three-component microseismometer was

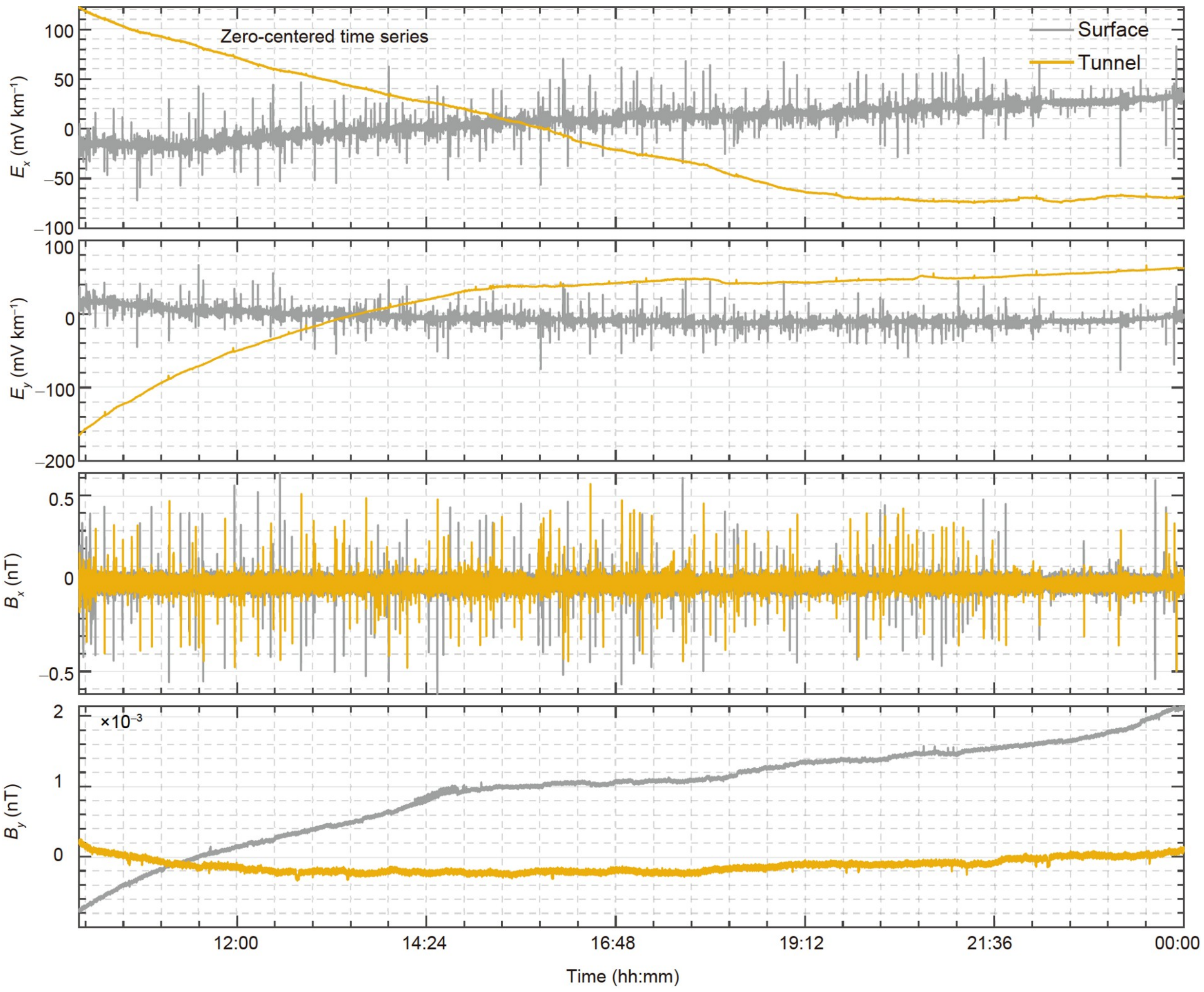

**Figure 8** Zero-centered MT time series at the surface (gray line) and the HDUL (yellow line). The horizontal electric/magnetic fields $E_x$, $E_y$, $B_x$, and $B_y$ are shown from top to bottom.

assembled by OMNI-2400 and PS-1 geophones. The sampling rate was set as 1 microsecond, and 12 hours of observations at three different locations were carried out at the surface and underground.

Figure 10 shows the time domain waveform of the three-component signals of underground Point 2010 and surface Point 2001. The three components of the same location are relatively stable and close. The comparisons between the surface and deep underground are obvious, and the ambient noises at the surface are significantly stronger than those underground, approximately 7.9–11.4 times greater. Comparing the PSD of the ambient noises, we find that, as illustrated in Figure 11, the underground noise mainly occupies the high-frequency band above 100 Hz, which is possibly caused by the running of the underground motors and pumps; the surface observation is mainly low-frequency interference below 50 Hz, and the PSD is more than two orders of magnitude stronger than the underground observation.

### *2.2.6 Seismic observation*

Four CMG-40TDE broadband translational seismometers and two R-2 rotational seismometers were employed in the surface and underground six-component seismic observations. Affected by the COVID-19 epidemic and instrument power supply, the translational seismometers recorded approximately 18 days of data, and the rotational seismometers worked for only 2 days.

Figure 12 presents the 24-hour translational seismic records at UTC on January 29, 2020, after preprocessing the data, including removing instrument responses. The noise at the surface is obviously stronger than that underground, as shown by the higher amplitude caused by human activities. Table 4 shows an earthquake event that occurred during the observation, and its three-component velocity waveform and amplitude spectrum are shown in Figure 13. In Figure 13, O is the time of the earthquake occurrence, and P and S represent the theoretical arrival times of the P- and S-waves based on the iasp91 model (Kennett and Engdahl, 1991).

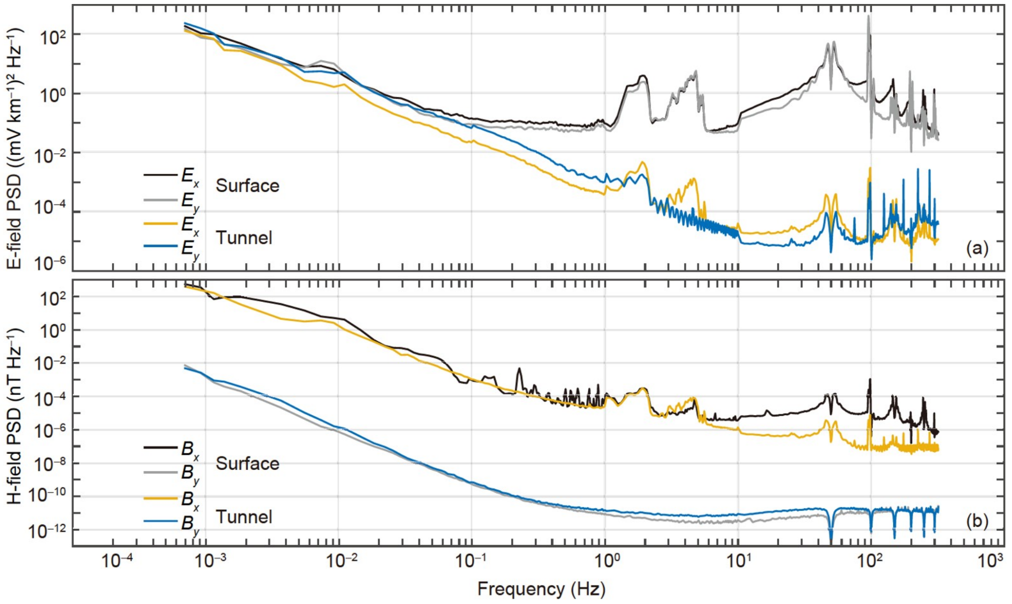

**Figure 9** Power spectral densities of the surface and underground MT data. (a) and (b) are the electric and magnetic fields, respectively. The colored lines delineate the results observed in the underground tunnel, in which the blue lines show the E-W field, and the yellow lines show the N-S field. The gray and black lines indicate the surface results, the black lines show the NS field, and the gray lines show the EW field.

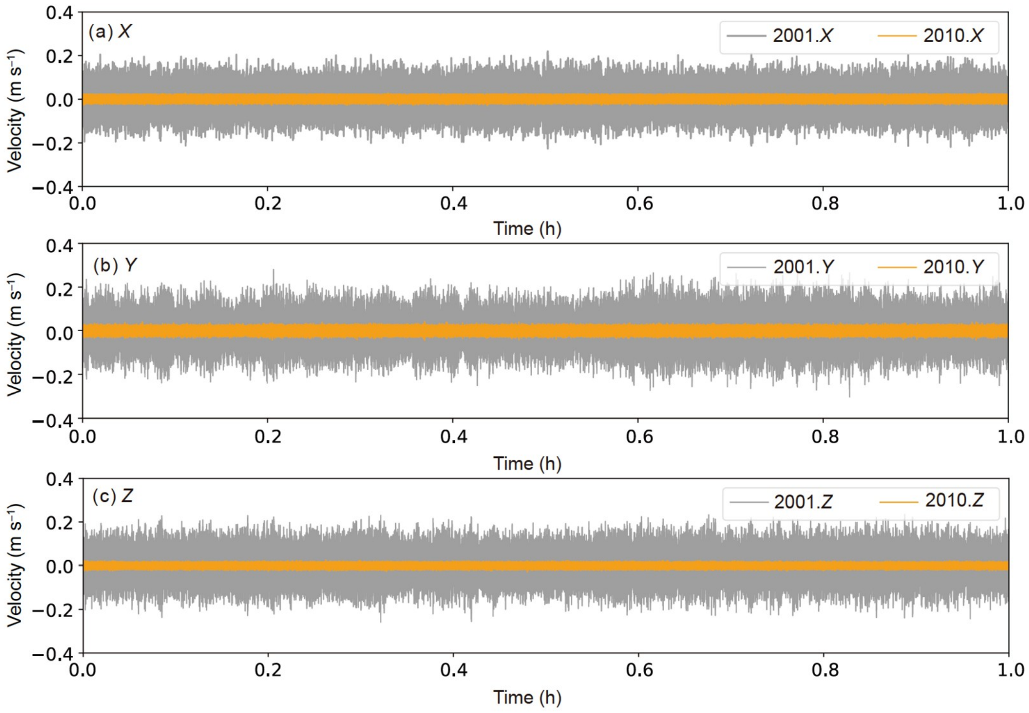

**Figure 10** Three-component microseismic waveforms observed at Point 2010 (underground) and Point 2001 (surface). (a)–(c) are *XYZ* three-component velocity.

Through comparison, we find that the earthquake signal recorded at the surface (N85) is stronger than that in the tunnel underground (N49), especially in the horizontal component, and their amplitude spectrum reveals the difference in different frequency bands: in the low-frequency band below 0.4 Hz, the amplitudes of the surface and subsurface observations are similar, while that in the frequency band of 0.4–0.8 Hz is different, which is speculated to reflect the influence of site effects caused by shallow loose deposits (Bravo and Sánchez-Sesma, 1990; Roten et al., 2013).

Due to the short observation duration and low instrumental accuracy, no obvious earthquake signal was identified in the records of the R-2 rotational seismometer. Figure 14 shows the rotational signals of 1 h recorded at the surface and un-

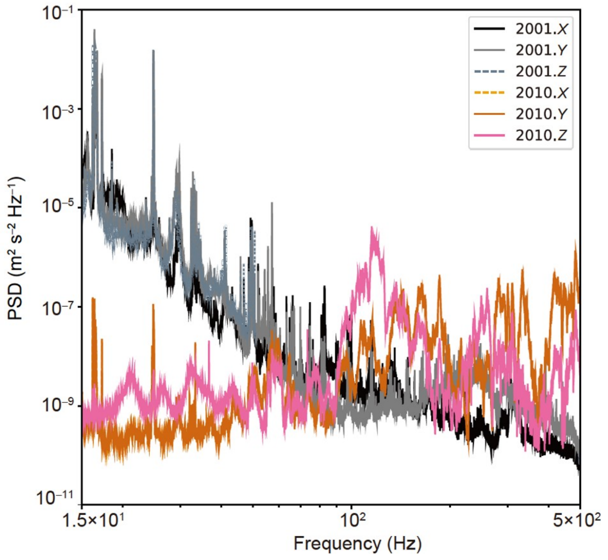

**Figure 11** PSD of ambient noise observed at Point 2010 (underground, the colored curves) and Point 2001 (surface, the dark curves).

derground stations, which mainly reflect the environmental noise and the self-noise of the instrument. Comparisons indicate that the energy of the surface rotation is slightly higher than that of the underground rotation. The PSD comparison in Figure 15 shows strong amplitude differences of the rotational components at the surface and underground. In the frequency band above 0.3 Hz, the $R_x$ and $R_z$ components at the surface are similar to those underground. However, in the low-frequency band below 0.3 Hz, rotational noises at the surface are stronger than those in the tunnel underground.

**Table 4** Time and location information of the *M*4.2 earthquake near the Yilan Sea, Taiwan Province, China[a)]

| Earthquake parameter | Value |
|---|---|
| Origin time (UTC) | 2020-01-31 06:09 |
| Magnitude (*M*) | 4.2 |
| Latitude | 24.86°N |
| Longitude | 122.05°E |
| Depth (km) | 10 |

a) Source: China Earthquake Networks Center (CENC) (https://news.ceic.ac.cn/)

The reason for the similar noise energy may be that the self-noise of the instrument is too high and fails to reflect the actual underground ambient noise. In future observations, higher-precision rotational seismometers should be tested to further verify the difference between the rotations at the surface and tunnel underground.

## 3. Discussion

In this paper, we report synchronous multi-physic field observation at the surface and subsurface deep underground in the HDUL, China, to investigate the noise level and confirm the superiority of the deep underground environment and its radiological safety. However, it is necessary to summarize the series of problems revealed in this observation and data excavation to promote deep underground scientific research.

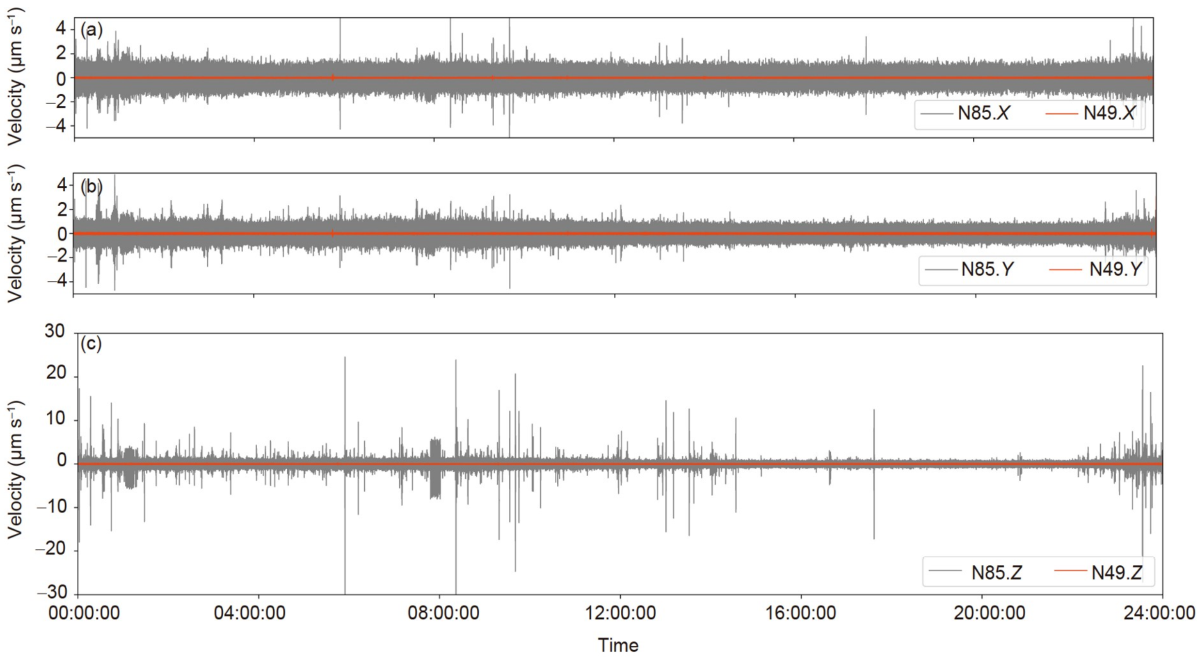

**Figure 12** Twenty-four-hour ambient noise records. N85 is the surface station, and N49 is the underground station; band filtering with $f$=1–24.5 Hz, UTC Time: 2020-01-29 00:00:00–24:00:00.

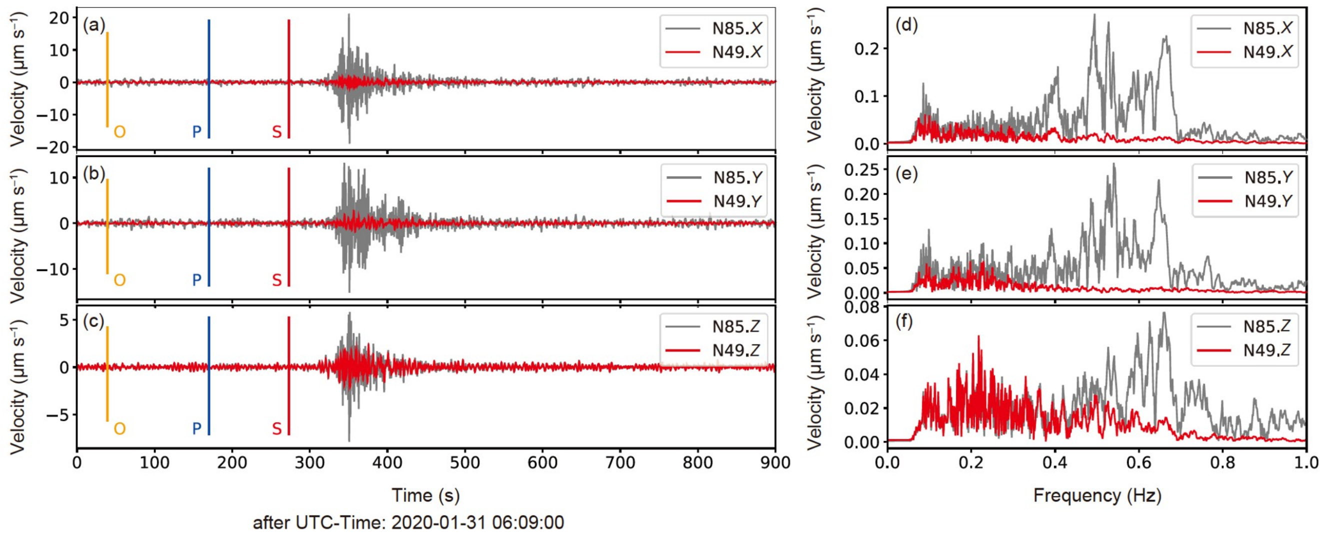

**Figure 13** Waveforms and amplitude spectra of the *M*4.2 earthquake near the Yilan Sea, Taiwan Province, China. (a)–(c) are the NS, EW and vertical components, respectively, and (d)–(f) are their amplitude spectra.

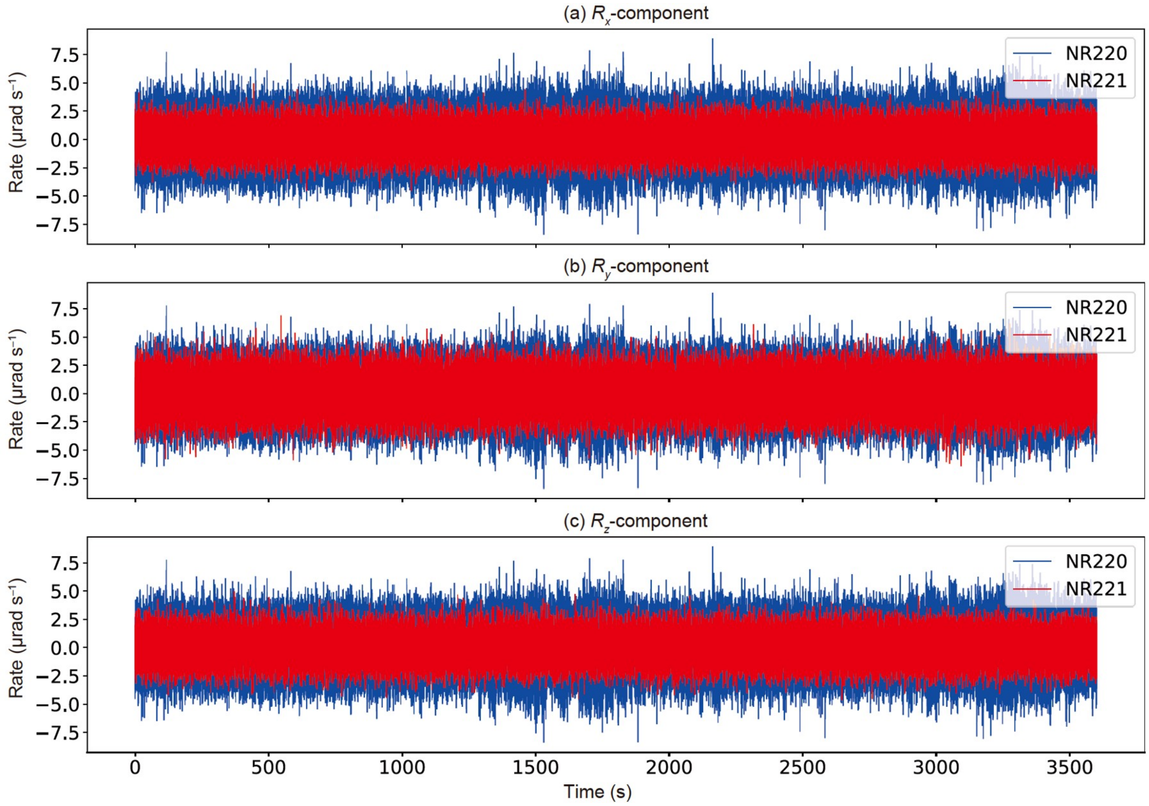

**Figure 14** Waveforms of rotational ambient noise at the surface and underground (N220 is surface station, N221 is underground station, same as Figure 15).

### 3.1 Adaptability and accuracy of the instruments

In this deep underground observation, general geophysical equipment designed for surface measurement was used. Apart from the power supply problem, which is relatively easy to overcome, the insufficiency of instruments for deep underground observation is one of the main problems exposed. These deficiencies are mainly due to the adaptability of the instruments, that is, the adjustments needed to provide timing and positioning in the absence of GPS signals, data transmission and long-term storage. For example, magnetometers, gravimeters, and seismometers all record data for a

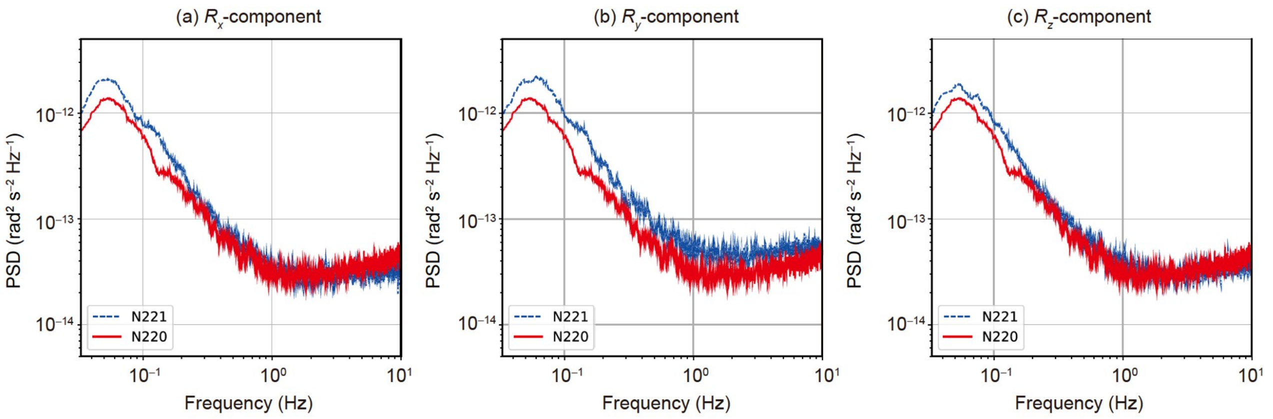

**Figure 15** PSD of rotational ambient noise observed at the surface and underground.

limited period due to insufficient battery capacity; rotational seismometers have problems of insufficient storage, causing data to be overwritten. Subsequent signal processing, such as manual timing, positioning, and time difference correction, solved some of these problems. Another important reason for the incomplete data is that the COVID-19 epidemic affected labor availability and power security. In the future construction of deep underground laboratories and the development of deep underground instruments, it is necessary to comprehensively consider at least two or more timing schemes, such as deeply wired GPS timing and NTP network timing, as well as accurate underground positioning, automatic data transmission and monitoring, large-capacity storage, power failure protection, etc.

The lack of precision and wide-frequency bands for these instruments is also an important problem. The scientific object of multi-physical deep underground observations is low-frequency (long/ultralong period) weak signals, which require high-sensitivity, wide-band, and high-precision instruments. For example, in the deep low cosmic ray environment, the $\gamma$ energy spectrometer and neutron detection showed serious shortcomings in the detection sensitivity and accuracy of the ultralow background environment; gravity, electromagnetic, and earthquake observation instruments all have insufficient period and sensitivity; instruments with electric field sensitivity reaching up to the nV–pV level and magnetic field up to the pT or fT level are urgently needed.

In addition, the self-noise of the instrument revealed by this observation needs further study. For example, the self-noise of the R-2 rotational seismometer may exceed the environmental noise. In later observations, fiber-optic rotational seismometers with higher precision can be used to further reduce the influence of instrument noise. Ensuring that the surface instruments meet the needs of high-precision, ultraquiet and ultraclean observations in the deep underground and that the stability of the instrument system and the response differences in different frequency bands meet the needs of high-precision and four-dimensional observations in the deep underground requires the calibration of sensor noise, instrument system noise, manufacturing environment, environmental noise, and many other issues.

### 3.2 Issues of observation systems and environment

Generally, radionuclide measurements take several days. Limited by field recording time, further measurement and analysis of $\gamma$ nuclides and total $\alpha$ and $\beta$ in underground water and coal/rock samples are not fully reported in this paper. In this radioactivity measurement, we focus on the accessibility and resident safety of the deep underground space.

To investigate the geomagnetic background of the deep tunnels, some typical and representative ferromagnetic locations and sites were specifically selected in the geomagnetic observation. The observations show that the ferromagnetic interference of the concrete tunnel obviously amplifies the geomagnetic field, and the steel-sealed chamber shields nearly half of the magnetic field. Therefore, long-term geomagnetic observations should be performed in open tunnels with fewer interference sources or reopened tunnels supported by nonmagnetic materials.

In the electromagnetic observation, due to the reinforced concrete structure of the underground tunnel, the burying of the instruments and the contact with the original rock did not provide ideal conditions, so the observation only lasted for 1 day. In future long-period electromagnetic observation, we need high-precision electromagnetic sensing equipment and locations with good contact with the original rock and far from electromagnetic interference. The solidification layout of electrodes and magnetic rods is also a prerequisite for future underground long-period electromagnetic observations.

In seismic observations, seismometers were placed on the concrete pavement on the east end of the underground main tunnel with poor coupling on the ground (Diaz et al., 2010; Ma X et al., 2019); the continuously operating ventilation system in the tunnel causes airflow disturbance, which also produces long-term disturbance to the records (Bonnefoy-Claudet et al., 2006). In future observations, partial reconstruction of the underground tunnel, construction of standard seismometer bases and wind proofing structures will further improve the accuracy.

Theoretically, combining surface, sky, and subsurface observations at different depths can reveal a significant four-dimensional gradient field, with which a series of meaningful geophysical inversion solutions can be given (Schmelzbach et al., 2018, 2019). Because of incomplete engineering preparation, environmental support and organization in this geophysical observation, effective 4D gradient recording was not achieved. In future observations, through appropriate engineering and environmental improvements, fiber-optic rotational seismometers, superconducting gravimeters and other instruments with higher precision should be used to further improve the accuracy of deep underground observations.

### 3.3 Advantages of deep underground observation in China

The regional location, covering thickness, surrounding rock lithology, depth (below 0 m of sea level), and various artificially introduced facilities will affect the observation environment of the underground laboratory (He et al., 2018). This geophysical observation in the HDUL is just a start and only provides local conclusions. Compared with underground laboratories that have been operating for many years worldwide, it can be inferred from our observations and preliminary analysis that deep underground observations can not only become an important supplement to ground-based and sky-based observations but also greatly improve the accuracy of observations and solve the problems faced by surface observations, such as interferences. These observations may also help to build an independent underground network, make high-precision observations of the physical gradient field possible, and provide high-quality data for the exploration of a series of fundamental scientific problems, such as the deep structure of the Earth and its dynamic processes. For example, true three-dimensional time-lapse geophysical fields can be obtained through joint observations in −848 m and −1042 m tunnels, enhanced by surface and air observations, which is beneficial for studying the spatial and temporal evolution of geophysical fields. With decades of rapid economic development in China, many underground spaces, such as metal mines and coal tunnels, have been built in the national land area. These deep underground spaces have many advantages, such as abundant wells, wide distribution, large underground space and good accessibility, which are important resources for building a unique deep and high-precision observation network in world.

It cannot be ignored that the facilities' maintenance and human activities deep underground will produce noise pollution, and the multiple physical fields will generate interference with each other. On the basis of high-precision observation, carrying out further research, including multi-physics interference shielding, signal and noise separation and noise control, is necessary for data insurance of high SNR, high reliability and high purity. Moreover, explorations of the high-accuracy multi-physical information will require advances in theoretical research and talent education in the future (Huang, 2005a, 2005b).

## 4. Conclusions

In this paper, only observations and preliminary analyses were introduced. Further data processing and joint research in the multi-physical field will be published in other articles. The following conclusions can be obtained from the first geophysical observation in the HDUL.

(1) Radioactive environment: The surrounding rocks and coal of the HDUL (−848 m) contain low levels of radionuclides, including uranium and radium, and the contents of uranium and radium in artificial wall coatings of tunnels and concrete supports underground are also relatively low. Therefore, the HDUL can be classified as an environment of low radioactivity (low radon concentration, low $X$-$\gamma$ dose rate and low neutron dose rate), which is suitable for long-term experiments and is also conducive to high-energy physics experiments and ultralow background cosmic research.

(2) Gravity observation: The surface and subsurface gravity noise levels show great differences at frequencies less than 1.7 mHz, especially in periods greater than 2 hours. The gravity noise level in the underground tunnel is nearly 2 orders of magnitude lower than that of the surface, indicating that the HDUL can provide excellent conditions for detecting weak gravity signals.

(3) Geomagnetic observation: The geomagnetic field recorded at the surface and deep underground tunnels shows obvious differences. The ferromagnetic material in the tunnel supporting structure generates a significant effect on the underground geomagnetic observations, and the steel-sealed chamber significantly weakens the normal geomagnetic field.

(4) Electromagnetic observation: Apparent differences in the PSD between the surface and underground tunnels suggested that the underground EM fields are significantly lower than the surface. The difference between electric and magnetic fields is nearly two and one order of magnitude for the frequency band above 10 Hz, respectively. The random EM noise of the high-frequency band is significantly weakened underground.

(5) Seismic observation: Compared with the surface, the underground environment has significantly lower vibration, which results in a higher data signal-to-noise ratio. The ventilation system, power system, elevator operation, and personnel movement in the deep environment bring unavoidable noises. Compared with long-period natural seismic signals, these noise signals are mainly high frequency and are easily filtered out. In the future, automatic identification and intelligent suppression of these noises will be essential for data mining and utilization. For the same earthquake event, the surface and subsurface three-component records show obvious differences in waveform and spectrum, and the site effect can easily be recognized.

In summary, a good ambient noise background can provide favorable conditions for high-precision 4D geophysical observation and then promote new ways to explore a series of fundamental problems in basic physics and Earth science, such as earthquake activity and the deep dynamic mechanism of the Tanlu fault zone, the dynamic process of different Earth spheres and it is their mechanisms of action with extraterrestrial bodies, the Lense-Thirring effect and spatial-temporal evolution, earthquake generation and the seismo-electric response mechanism.

**Acknowledgements** *We thank Huaihe Energy Company Limited for providing help with these deep underground observations and for providing much support in deep underground security. Professor Liu Xiangdong at Huazhong University of Science and Technology put forward valuable opinions on the writing of this article. Many teachers and students from China University of Geosciences (Beijing), Innovation Academy for Precision Measurement Science and Technology, CAS, and East China University of Technology participated in this joint observation. This research was supported by the National Natural Science Foundation of China (Grant Nos. 62127815, 42150201, U1839208).*

**Open Access** This article is licensed under a Creative Commons Attribution 4.0 International License, which permits use, sharing, adaptation, distribution and reproduction in any medium or format, as long as you give appropriate credit to the original author(s) and the source, provide a link to the Creative Commons licence, and indicate if changes were made. The images or other third party material in this article are included in the article's Creative Commons licence, unless indicated otherwise in a credit line to the material. If material is not included in the article's Creative Commons licence and your intended use is not permitted by statutory regulation or exceeds the permitted use, you will need to obtain permission directly from the copyright holder. To view a copy of this licence, visit http://creativecommons.org/licenses/by/4.0/.

## Electronic Supplementary Material

Appendix
