# Supplementary material for: Observation and research of deep underground multi-physical fields—Huainan −848 m deep experiment
Source: Sci China Earth Sci. 2022 Dec 19;66(1):54–70. doi: 10.1007/s11430-022-9998-2 (PMC9782270; doi:10.1007/s11430-022-9998-2)
Supplement: Supplementary file 1 — Appendix [file 11430_2022_9998_MOESM1_ESM.pdf]

Appendix

Table S1 Part of the earthquake catalog during the observation period [a)]

Event	Date and time (UTC+8)	*M*	Latitude (°N)	Longitude (°E)	Depth (km)	Reference location
1	2020-01-19 11:22:33	4.1	21.73	121.76	10	Taitung County, Taiwan Province, China
2	2020-01-20 04:17:11	3.7	23.86	116.01	10	Xingning City, Meizhou City, Guangdong Province, China
3	2020-01-20 04:28:00	2.7	23.86	116.01	10	Xingning City, Meizhou City, Guangdong Province, China
4	2020-01-22 15:08:33	4.5	31.66	103.13	13	Li County, Aba Prefecture, Sichuan Province, China
5	2020-01-23 05:26:23	3.5	23.35	101.9	8	Honghe County, Honghe Prefecture, Yunnan Province, China
6	2020-01-23 22:57:28	3.1	31.67	103.13	9	Li County, Aba Prefecture, Sichuan Province, China
7	2020-01-27 18:55:47	3	28.83	105.11	8	Jiang'an County, Yibin City, Sichuan Province, China
8	2020-01-28 20:43:45	3.5	33.05	121.38	14	Waters of Dongtai City, Yancheng City, Jiangsu Province, China
9	2020-01-29 07:39:29	5.3	27.16	126.6	10	East China Sea
10	2020-01-29 22:00:43	3	32.08	103.49	16	Mao County, Aba Prefecture, Sichuan Province, China
11	2020-01-30 04:20:14	3.4	28.35	104.95	11	Changning County, Yibin, Sichuan Province, China
12	2020-01-31 14:09:39	4.2	24.86	122.05	10	Waters near Yilan County, Taiwan Province, China
13	2020-02-02 14:35:49	2.9	32.41	105.11	14	Qingchuan County, Guangyuan City, Sichuan Province, China
14	2020-02-03 00:05:41	5.1	30.74	104.46	21	Qingbaijiang District, Chengdu, Sichuan Province, China
15	2020-02-03 02:15:03	2.9	32.02	104.51	14	Beichuan County, Mianyang City, Sichuan Province, China
16	2020-02-03 23:55:51	3.9	28.48	104.73	12	Gong County, Yibin City, Sichuan Province, China
17	2020-03-30 16:20:59	4	40.14	111.85	14	Horinger County, Hohhot City, Inner Mongolia, China
18	2020-04-05 14:31:58	3.2	37.85	106.27	13	Litong District, Wuzhong City, Ningxia Province, China
19	2020-04-06 15:01:41	3	28.21	105.07	8	Xingwen County, Yibin City, Sichuan Province, China
20	2020-04-09 07:49:18	4.4	24.27	121.95	24	Waters off Hualien County, Taiwan Province, China
21	2020-04-09 09:14:25	3.3	28.14	104.72	8	Junlian County, Yibin City, Sichuan Province, China
22	2020-04-11 00:44:56	5.9	20.35	122.15	150	Philippine Islands Region
23	2020-04-12 19:36:39	4.2	24.03	122.29	24	Waters off Hualien County, Taiwan Province, China
24	2020-04-13 13:17:56	3.2	38.77	116.18	8	Renqiu City, Cangzhou City, Hebei Province, China
25	2020-04-27 09:37:12	3.3	28.18	104.78	9	Gong County, Yibin City, Sichuan Province, China
26	2020-05-03 11:24:40	5.4	23.29	121.6	40	Taitung County, Taiwan Province, China
27	2020-05-03 19:54:26	6	31.37	128.7	10	Waters near Kyushu Island, Japan

a) From China Earthquake Networks Center (CENC): http://www.ceic.ac.cn/
